# Transcriptome analysis reveals key drought-stress-responsive genes in soybean

**DOI:** 10.3389/fgene.2022.1060529

**Published:** 2022-11-28

**Authors:** Mingqian Li, Hainan Li, Anni Sun, Liwei Wang, Chuanyou Ren, Jiang Liu, Xining Gao

**Affiliations:** ^1^ College of Agronomy, Shenyang Agricultural University, Shenyang, China; ^2^ Liaoning Key Laboratory of Agrometeorological Disasters, Shenyang, China

**Keywords:** drought stress, soybean, transcriptome, WGCNA, metabolic pathway, yeild

## Abstract

Drought is the most common environmental stress and has had dramatic impacts on soybean (*Glycine* max L.) growth and yield worldwide. Therefore, to investigate the response mechanism underlying soybean resistance to drought stress, the drought-sensitive cultivar “Liaodou 15” was exposed to 7 (mild drought stress, LD), 17 (moderate drought stress, MD) and 27 (severe drought stress, SD) days of drought stress at the flowering stage followed by rehydration until harvest. A total of 2214, 3684 and 2985 differentially expressed genes (DEGs) in LD/CK1, MD/CK2, and SD/CK3, respectively, were identified by RNA-seq. Weighted gene co-expression network analysis (WGCNA) revealed the drought-response TFs such as WRKY (*Glyma.15G021900, Glyma.15G006800*), MYB (*Glyma.15G190100, Glyma.15G237900*), and bZIP (*Glyma.15G114800*), which may be regulated soybean drought resistance. Second, *Glyma.08G176300 (NCED1), Glyma.03G222600 (SDR), Glyma.02G048400 (F3H), Glyma.14G221200 (CAD), Glyma.14G205200 (C4H)*, *Glyma.19G105100 (CHS), Glyma.07G266200 (VTC)* and *Glyma.15G251500 (GST),* which are involved in ABA and flavonoid biosynthesis and ascorbic acid and glutathione metabolism, were identified, suggesting that these metabolic pathways play key roles in the soybean response to drought. Finally, the soybean yield after rehydration was reduced by 50% under severe drought stress. Collectively, our study deepens the understanding of soybean drought resistance mechanisms and provides a theoretical basis for the soybean drought resistance molecular breeding and effectively adjusts water-saving irrigation for soybean under field production.

## 1 Introduction

Soybean (*Glycine* max L.), as one of the most important oil crops with significant economic value, has been cultivated worldwide. In addition to its macronutrients and minerals, soybean has many positive effects on human health due to its contents of oil, protein and isoflavones (Sakai and Kogiso, 2008; Choudhary and Tran, 2011; He and Chen, 2013). However, drought is the most common environmental stress encountered by plants under the current situation of climate change, which has had dramatic impacts on plant growth and crop yield (Barnab´as et al., 2008; Qin et al., 2011; Lesk et al., 2016), such as up to a 40% reduction in soybean yield (Stacey et al., 2004; Fahad et al., 2017). Consequently, the molecular mechanisms and molecular breeding of drought tolerance in soybean remain to be explained (Lawlor, 2013).

Drought stress has negative effects at the physiological, developmental, and molecular levels in plants**,** including photosynthesis inhibition, reactive oxygen species (ROS) generation, and cellular tissue and membrane damage (Xu et al., 2010; Golldack et al., 2014; Zhu, 2016; Anjum et al., 2017). Thus far, a number of studies have suggested that plants use multiple physiological and molecular strategies in response to drought stress. For instance, they rapidly accumulate osmotic regulators (proline and soluble sugar), a process crucial for plant drought resistance **(** Dien et al., 2019; La et al., 2019)**.** Furthermore, the increase in superoxide dismutase (SOD) and peroxidase (POD) activities enhances vitamin C production and glutathione metabolism, playing a key role in avoiding drought damage (Sun et al., 2020; Zhang et al., 2020). In addition, plants can activate drought stress defense though the altered expression of related genes, such as *4CL5* and *F5H1* involved in flavonoid pathway and lignin biosynthesis, which results in increased lignin content and subsequent drought tolerance (Xu et al., 2020; Sun et al., 2022).

Plant hormones responding to abiotic and biotic stresses play significant roles in plant growth and development. It has been well documented that drought stress can cause the biosynthesis and signal transduction of various plant hormones, especially abscisic acid (ABA) (Khan et al., 2015; Vishwakarma et al., 2017). Significantly, the NCED gene encodes 9-cis-epoxycarotenoid dioxygenase, which can increase abscisic acid content and induce drought stress related genes, stomatal closure and other physiological processes in plants (Chimungu et al., 2014; Huang et al., 2019). The ABA responsive elements binding factor (ABF) has been reported to regulate the expression of drought-responsive genes and enhance enzyme activity to maintain plant resistance to drought stress (Kerr et al., 2018).

Various omics analyses, such as genomics, transcriptomics, proteomics, and metabolomics, have gained insight into plant responses to abiotic stresses (Basim et al., 2021). Due to technological advancements and reduced cost, RNA sequencing, has become one of the most effective methods for evaluating the interaction between plants and abiotic stresses (Zhang and Song, 2017; Xuan et al., 2022). Based on transcriptomics analysis, many transcription factors (TFs) have been detected in plants associated with drought stress, including WRKY, MYB and DREB (Lindemose et al., 2013; Wang et al., 2016), AP2/ERF (Xie et al., 2019), bZIP (Kang et al., 2019), and NAC (Thirumalaikumar et al., 2018). In addition, the mitogen-activated protein kinase (MAPK) signaling pathway and the Ca^2+^ signaling pathway have been found to be enriched under drought stress based on KEGG analysis (Zhao et al., 2020; Liu et al., 2022).

Soybean is known to be highly sensitive to water deficit; hence, an adequate water supply is crucial for its growth and development to achieve high primary production (Buezo et al., 2019; Yu et al., 2020). However, there can be a certain degree of drought stress in soybean, which has no significant effects on soybean yield due to plant growth compensation after rehydration (Hao et al., 2010; Liu et al., 2012; Xue et al., 2013). Previous studies have shown that the flowering stage of soybean is the most sensitive period for drought stress (Meckel et al., 1984). Therefore, to investigate the response mechanism of soybean under varying degrees of drought, the drought-sensitive cultivar “Liaodou 15” was exposed to different levels of drought stress at the flowering stage by gradually decreasing the amount of irrigation at different levels and durations. Subsequently, physiological evaluation and transcriptomic analyses in soybean leaves under drought stress were performed. Then, WGCNA based on the transcriptome data and physiological indices was performed to investigate the function of TFs and identify genes of several key pathways in soybean response under drought stress. Meanwhile, we analyzed the crop yield of soybean after rehydration in the harvesting period. Collectively, the aim of this work was to elucidate the mechanism underlying the response of soybean to drought stress, provide a theoretical basis for the molecular breeding of drought resistance, and effectively adjust water-saving irrigation for soybean production under field conditions.

## 2 Materials and methods

### 2.1 Plant materials and equipment

The soybean, the drought-sensitive cultivar “Liaodou 15”, was grown in the scientific observation and experimental station of crop cultivation in Northeast China, Ministry of Agriculture and Rural Affairs, P. R. China, located at Shenyang Agricultural University (123.53°E, 41.73°N, Shenyang, China).

The area has a brown soil type, and the soil capacity at the time was 30%. The basic soil fertility data are shown in Supplementary Table S1. The experimental equipment included a sliding plastic film rain shelter with a reinforced steel frame, which was used on rainy days, and a soil moisture and temperature sensor buried approximately 30 cm deep in the soil (SMTS-II-485, China). This study applied a drip system to control the amount of water released during each treatment to ensure uniform irrigation. Except for the water control, the other cultivation measures were the same as in standard procedures during the entire growth period.

### 2.2 Experimental design

There were three drought stress treatments in the experiment and three repetitions for the control. This study adopted a randomized block design. Each drought treatment had three plot replicates, for a total number of 12. The size of each plot was 2 × 3.6 m. A total of 108 soybean plants were grown in each plot. The relative soil water content was measured by a soil moisture and temperature sensor (SMTS-II-485, China). Before flowering, all plots should maintain the same soil moisture conditions and sufficient water content.

For drought treatments, soybean plants at the early flowering stage were continuously subjected to drought stress. 1) Water was continuously withheld from the first group for 7 days. As a result, the soybean leaves appeared curled (mild, the soil moisture content was 24.3%, LD). 2) Water was continuously withheld from the second group for 17 days, wilting and curling (moderate, the soil moisture content was 20.6%, MD). 3) Water was continuously withheld from the third group for 27 days, severe wilting and curling (severe, the soil moisture content was 16.9%, SD). 4) The control leaves of each treatment with the same developmental stage were grown in soil with 30% relative water content (CK1, CK2, and CK3), which remained green, fully expanded and healthy. After the drought stress period was completed, rehydration was performed with the control level on the same day, and this was maintained until harvest. Meanwhile, the top two to three leaf samples with each drought stress treatment and corresponding controls were collected on the day of completing the drought experiment, and the samples were quickly frozen in liquid nitrogen, then stored at −80°C until measurement. The leaves of each soybean sample type (*n* = 30) were used for the evaluation of physiological indices and transcriptome analysis. Soybean seeds were used for yield measurement. All experiments were performed with at least three biological replicates.

### 2.3 Determination of physiological indices

The collected soybean leaves under drought stress were examined for seven physiological indices. The contents of soluble sugar (cat no. YX-W-B602), soluble protein (cat no. YX-W-C202), chlorophyll (YX-W-A304), proline (ca no. YX-W-A605) and malondialdehyde (MDA, cat no. YX-W-A401), as well as the SOD (cat no. YX-W-A500 -WST-8) and CAT (cat no. YX-W-A501) activities were determined according to the instructions of the physiological index assay kit provided by Sinobestbio Biotechnology Co., Ltd. (Shanghai, China). Each sample was used for three technical replications.

### 2.4 Soybean RNA-seq analysis

Leaves of soybean were collected at 7, 17, and 27 d after drought stress with three biological replicates. Transcriptome sequencing was performed by Genedenovo Biotechnology Co., Ltd (Guangzhou, China). Briefly, total RNA was extracted and checked for purity and integrity using a NanoDrop 2000 (Thermo Scientific, Waltham, MA, United States) and the RNA Nano 6000 Assay Kit of the Bioanalyzer 2100 System (Agilent Technologies, Santa Clara, CA, United States), respectively. The qualified RNA was prepared for the construction of cDNA libraries and sequenced using an Illumina sequencing platform. The clean reads were mapped to the soybean genome (https://phytozome-next.jgi.doe.gov/info/Gmax_Wm82_a4_v1) by HISAT2 tools (Langmead et al., 2009; Kim et al., 2015). The raw sequencing data generated from this study were archived in NCBI SRA (http://www.ncbi.nlm.nih.gov/sra) with the BioProject accession number PRJNA852689. A power analysis for sequencing depth was calculated by RNASeqPower (https://doi.org/doi:10.18129/B9.bioc.RNASeqPower), which uses RNA-seq data analysis to examine transcription patterns. Differential expression analysis of soybean plants under drought stress was performed using DESeq2 (Love et al., 2014). DEGs with |log2FC| > 1 and *p*-values ≤ 0.05 were retained and considered significantly upregulated or downregulated groups, respectively. The expressed gene function annotations were conducted by the Gene Ontology (GO) database. All molecular pathways were explored by the Kyoto Encyclopedia of Genes and Genomes (KEGG).

### 2.5 Weighted gene co-expression network analysis

The R package “weighted gene co-expression network analysis” (WGCNA) was used to identify the network of genes from the transcriptome data and seven physiological indices. The DEGs were divided into different modules marked with different colors based on similar expression patterns. Combined with the changes in seven physiological indices, the DEGs associated with drought resistance were analyzed in the modules using the significant correlation coefficient, which was performed by KEGG and GO enrichment analysis.

### 2.6 Validation of RNA‒seq data by qRT‒PCR analysis

Total RNA from soybean leaves was extracted by an ultrapure RNA kit (Cat#CW0581, CWbio. Co. Ltd., Beijing, China) and treated with DNase I (DNA free, Takara, Dalian, China). The concentration and quality of total RNA was checked by a NanoDrop device (Thermo, Fisher Scientific). First-strand cDNA synthesis was carried out from 1 μg of treated total RNA using PrimeScript™ RT Master Mix (Takara, Dalian, China) in a total volume of 20 μl.

Relative gene expression was quantified by qRT‒PCR using SuperReal PreMix Plus (SYBR Green) (Takara, Dalian, China) according to the manufacturer’s protocol on an ABI PRISM 7500 sequence detection system (Applied Biosystems, Thermo Fisher Scientific, United States). The gene-specific primers for real-time quantitative PCR (qRT‒PCR) were designed using the NCBI online tool Primer-blast (https://blast.ncbi.nlm.nih.gov/Blast.cgi) and are listed in Supplementary Table S2. The amplification program was as follows: one cycle of 30 s at 95 °C, followed by 45 cycles of 5 s at 95°C, 15 s at 60°C and 72°C for 15 s. The soybean gene *GmUKN1 (Glyma12g02310)* served as the internal reference gene and the 2^−ΔΔCt^ method was used to analyze relative changes of gene expression (Livak and Schmittgen, 2001; Lu et al., 2016). Three biological and three technical replicates were included in the qRT‒PCR analysis.

### 2.7 Soybean yield measurement

After soybean ripening, thirty soybean plants were randomly selected from each plot and harvested in the laboratory. The seed weight and soybean yield in each plot were investigated.

### 2.8 Statistical analysis

All measurements were repeated at least three times. The data are presented as the mean values ± standard deviations (SD) and subjected to analysis of variance (ANOVA). Multiple comparisons were conducted by Duncan’s multiple range test at *p* < 0.05 using the SPSS statistics program 18.0. The differences between the physiological index and gene expression in response to drought stress were considered statistically significant at *p*-values < 0.05. The charts were drawn by Excel 2010 (Microsoft, Redmond, WA, United States) and TBtools (Chen et al., 2020).

## 3 Results

### 3.1 Physiological responses of soybean under drought stress

**FIGURE 1:**
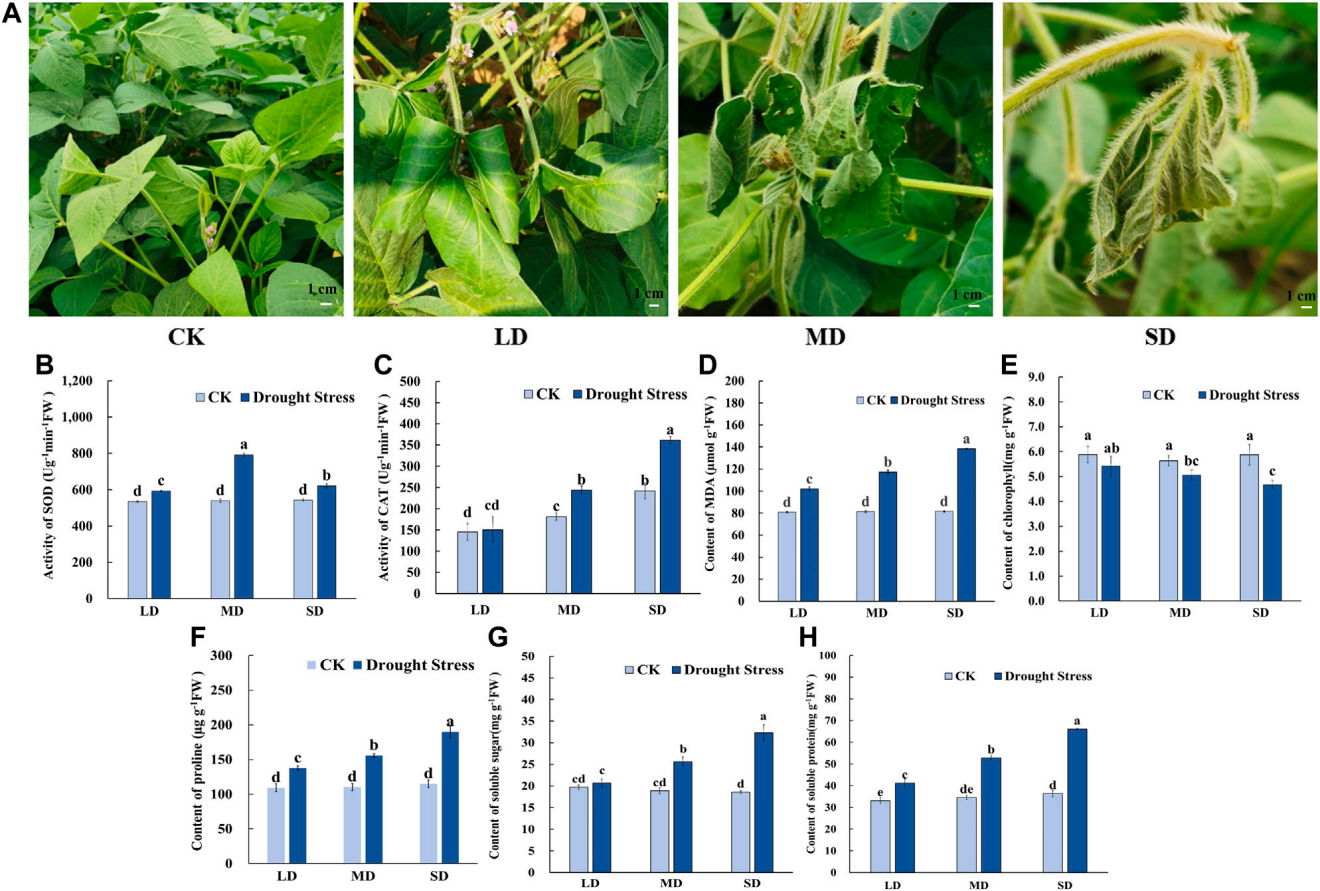
Phenotypic characteristics and physiological properties of soybean under drought stress. **(A)** Phenotypic characteristics of soybean under drought stress. **(B)** The activity of SOD. **(C)** The activity of CAT. **(D)** The content of MDA. **(E)** The content of chlorophyll. **(F)** The content of proline **(G)** The content of soluble sugars. **(H)** The content of soluble protein. Three biological replicates were performed and the data represent the means ± SDs. Different letters represent statistically significant differences (*p* < 0.05).

To gain insights into the mechanism of the drought stress response in soybean at the flowering stage, we triggered a controlled water deficit in the experimental application to simulate different drought levels. Plants in each treatment group to a different level of soil moisture content are shown in Supplementary Figure S1, which was measured by a soil moisture and temperature sensor (SMTS-II-485, China). When exposed to drought stress for 7 d, the soybean leaves showed slightly curled edges, corresponding to mild drought stress (24.3% soil moisture content, LD). After 17 d of water deprivation, moderate drought stress was attained (20.6% soil moisture content, MD), and some soybean plants turned curved and partially yellowed surfaces with signs of water loss, while control plants remained healthy. Moreover, following 27 days without watering, almost all soybean plants appeared severe wilting and curling, indicating severe drought stress-induced damage (16.9% soil moisture content, SD), whereas the leaves of the control remained green and fully expanded (Figure 1A). Accompanied by changes in leaf phenotype, the chlorophyll content steadily decreased with decreasing soil moisture content from 30% to 16.9% and was markedly lower than that under control conditions (Figure 1E). The MDA content peaked in the SD treatment at 138.4 μmol/g, MDA content in SD treatment did differ significantly among the rest of the treatments, and each drought treatment was significantly higher than that of the control (Figure 1D).

Meanwhile, for the antioxidant enzyme activity in soybean leaves, the SOD activity tended to increase first and then decrease as drought time increased from 7 d to 27 d in the soybean (Figure 1B). The SOD activity of soybean in the MD treatment reached a maximum of 791.59 U/g min at 17 days, whereas significant differences were noted among the other treatments. The CAT activity was not significantly different under the LD condition compared to the control, and CAT activity was significantly increased (Figure 1C). Furthermore, for osmotic adjustment in soybean leaves, the proline content increased following the LD, MD and SD treatments. Soybean reached a maximum proline content in the SD treatment of 193.29 μg/g (Figure 1F). Consistent with this, the contents of soluble sugars and soluble protein were not significantly different under LD compared with the control, whereas soluble sugars content of 25.64 mg/g, and 32.33 mg/g and soluble protein content with 52.84 mg/g, 66.06 mg/g in soybean for the MD and SD treatments were noted compared with their control (Figures 1G,H).

In short, these results indicated that in soybeans exposed to varying degrees of drought stress due to prolonged water control, osmotic regulatory substances were increased and antioxidant enzyme activity was enhanced to maintain plant growth and development.

### 3.2 RNA-seq data revealed differentially expressed genes in soybean after drought treatments

To elucidate the mechanism of the response of differentially expressed genes in drought stress in soybean, RNA-seq was performed in soybean leaves subjected to different levels of drought stress. By comparing reads to the soybean genome, the genomic alignment of each sample was obtained, and the alignment rate was approximately 93% (Supplementary Table S3). Based on three biological replicates under control and different drought conditions, we performed a principal component analysis (PCA). The first two principal components PC1 and PC2 accounted for 79.5% and 10.4%, respectively (Supplementary Figure S2A). These results indicated that there were different gene expression patterns between the soybeans under different levels of drought stress (Supplementary Figure S2B). In addition, the power analysis for sequencing depth was performed to identify DEGs (Supplementary Table S4). The statistical power of this experimental design, calculated in three drought treatments (CK1 vs. LD, CK2 vs. MD, and CK3 vs. SD), was 0.7655, 0.7654 and 0.7656, respectively (Supplementary Table S4).

**FIGURE 2:**
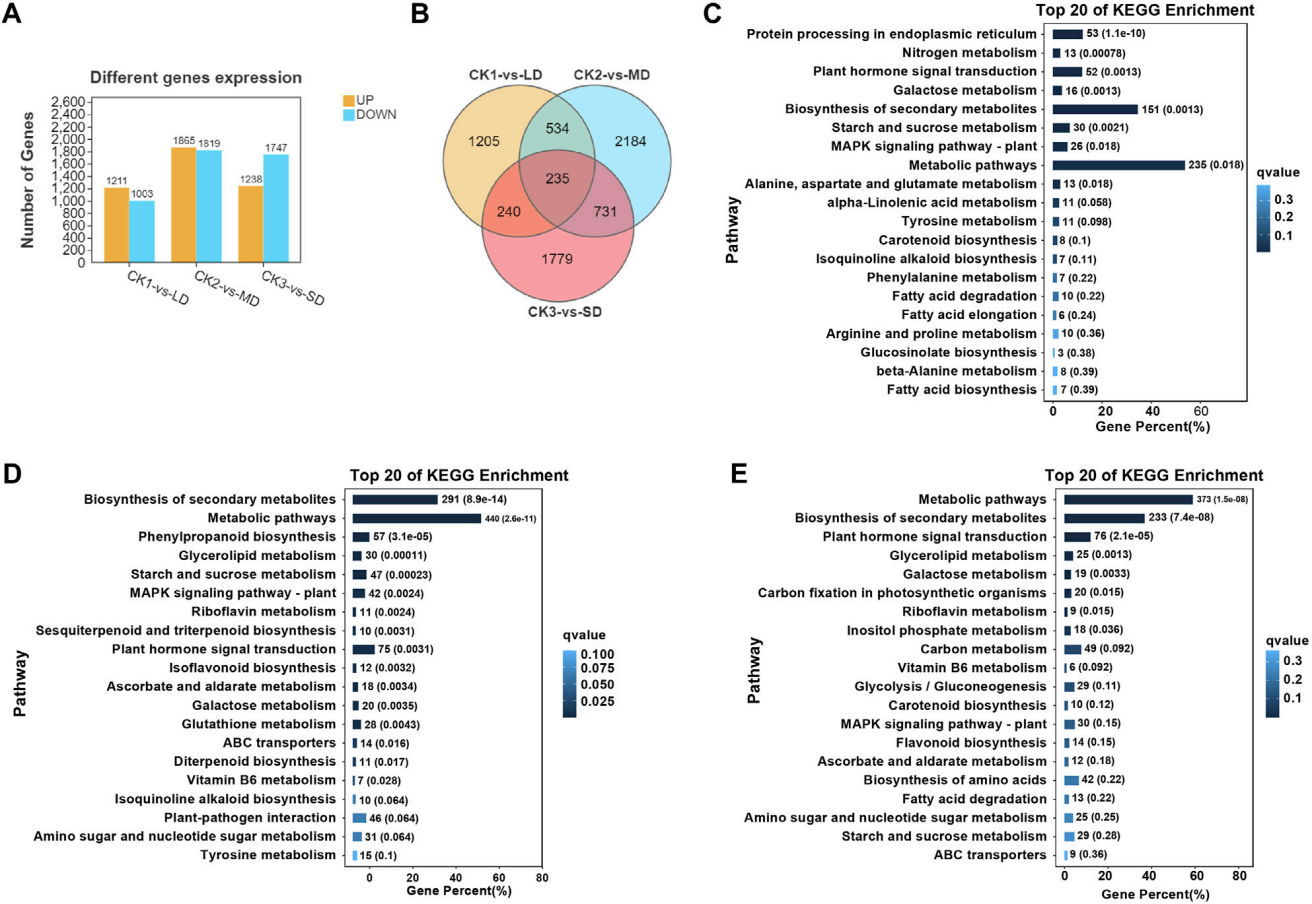
Transcriptional analysis of soybean under various drought stresses. **(A)** The numbers of DEGs. **(B)** Venn diagrams show the overlapping DEGs between the three drought stress. **(C)** The top20 enriched of KEGG pathways in soybean under LD/CK1. **(D)** The top20 enriched of KEGG pathways in soybean under MD/CK2. **(E)** The top20 enriched of KEGG pathways in soybean under SD/CK3.

Subsequently, the analysis of differentially expressed genes was performed in soybean leaves exposed to three levels of drought stress and compared with the control, which could identify the significantly changed genes regulated by drought stress (Figure 2). There were 1211 upregulated and 1003 downregulated genes in CK1 vs. LD, and 1865 upregulated and 1819 downregulated DEGs in CK2 vs. MD. A total of 2985 DEGs were identified in leaves exposed to SD, including 1238 upregulated and 1747 downregulated genes (Figure 2A). Furthermore, the Venn diagrams showed that 235 DEGs were common in soybeans under three drought treatments (CK1 vs. LD, CK2 vs. MD, and CK3 vs. SD) (Figure 2B)**.** The gene expression of 235 common DEGs in soybean under three levels of drought stress is shown in Supplementary Table S5 **.**

### 3.3 Functional enrichment analysis of DEGs by GO and KEGG

In order to analyze the differentially expressed genes in soybean in response to drought stress, gene ontology (GO) and KEGG analyses were performed, which highlighted the major functions of genes induced by drought stress. The GO enrichment analysis was conducted with division into biological process (BP), molecular function (MF) or cellular component (CC) terms. The majority of drought-responsive genes in soybean were significantly enriched regarding BP terms, including ‘cellular process’ and “metabolic process”, as well as MF categories, including ‘binding’ and “catalytic activity”. Several CC terms were mainly characterized as ‘cell’, “cell part”, “organelle”, and “membrane” (Supplementary Figures S3–5). The number of differentially expressed genes (up- or downregulated) in each pathway under mild drought was less than 800, while those under MD and SD were all higher than 1000. These results indicated that DEGs were related to metabolic pathways or other regulatory networks that respond to drought stress.

On the basis of the obtained transcriptome data, we specified the top 20 KEGG pathways under drought stress. The KEGG analysis showed that three pathways were significantly enriched (false discovery rate ≤5%) under varying degrees of drought stress, including starch and sucrose metabolism, plant hormone signal transduction, and MAPK signaling pathways (Figures 2C–E). Interestingly, significant enrichment of arginine and proline metabolism was detected only under mild drought stress (Figure 2C). MD and SD stress stimuli trigger the ascorbate and aldarate metabolism pathways. However, glutathione metabolism and flavonoid biosynthesis pathways were differentially expressed between MD and SD stress (Figures 2D,E). This observation indicates that the enrichment of antioxidant and secondary metabolite pathways are considered typical of the response of soybean to different drought stress treatments.

### 3.4 Co-expression network analysis of DEGs in soybean leaves

**FIGURE 3:**
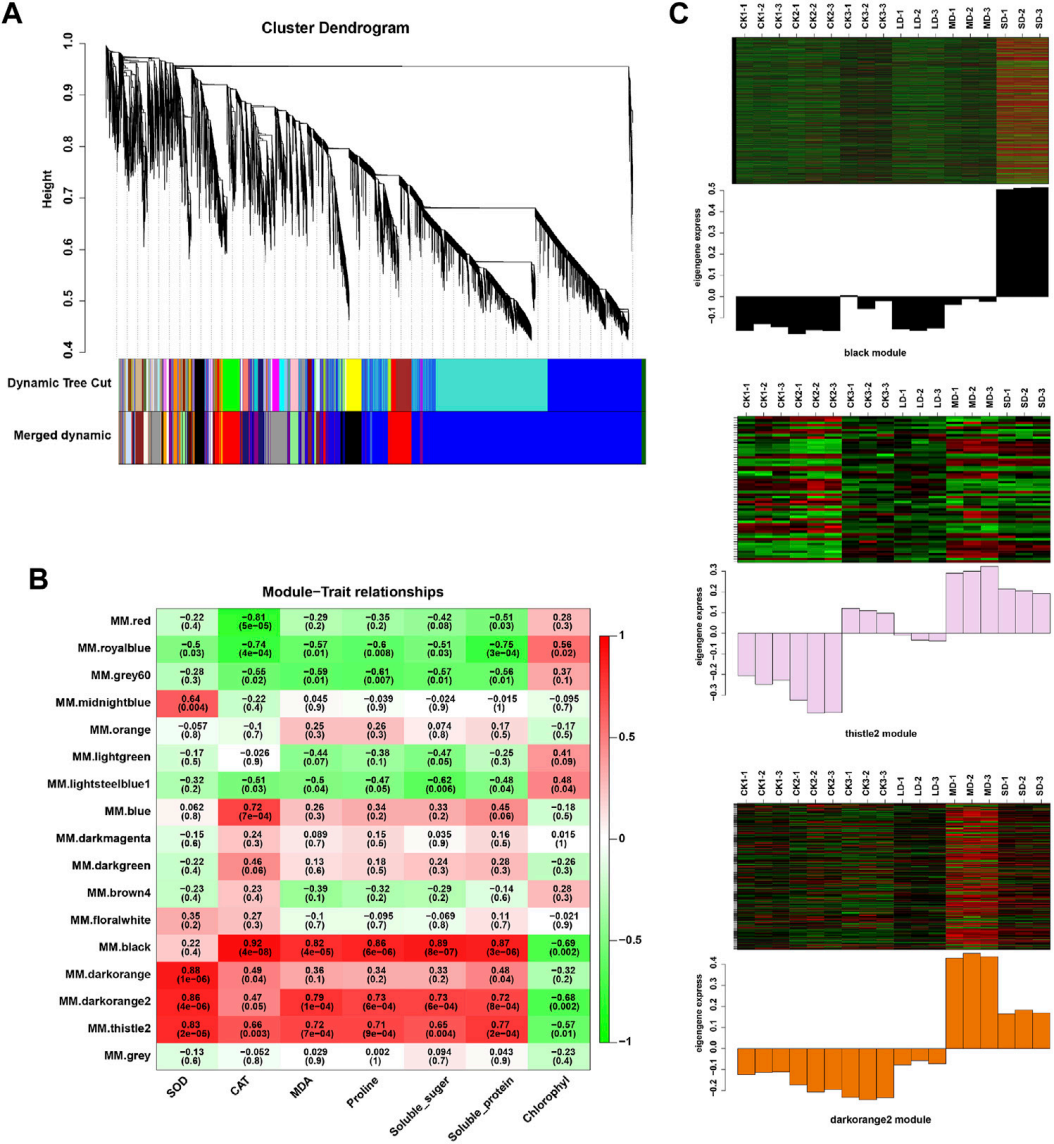
WGCNA of DEGs in soybean under various drought stresses. **(A)** Seventeen co-expression modules shown by a hierarchical cluster tree. **(B)** The correlation analysis between modules and physiological traits. **(C)** Heatmaps indicate the expression patterns of genes in the black, drakorange2, and thistle2 modules. The samples were CK1, CK2, CK3, LD, MD, and SD with three biological replicates.

**FIGURE 4:**
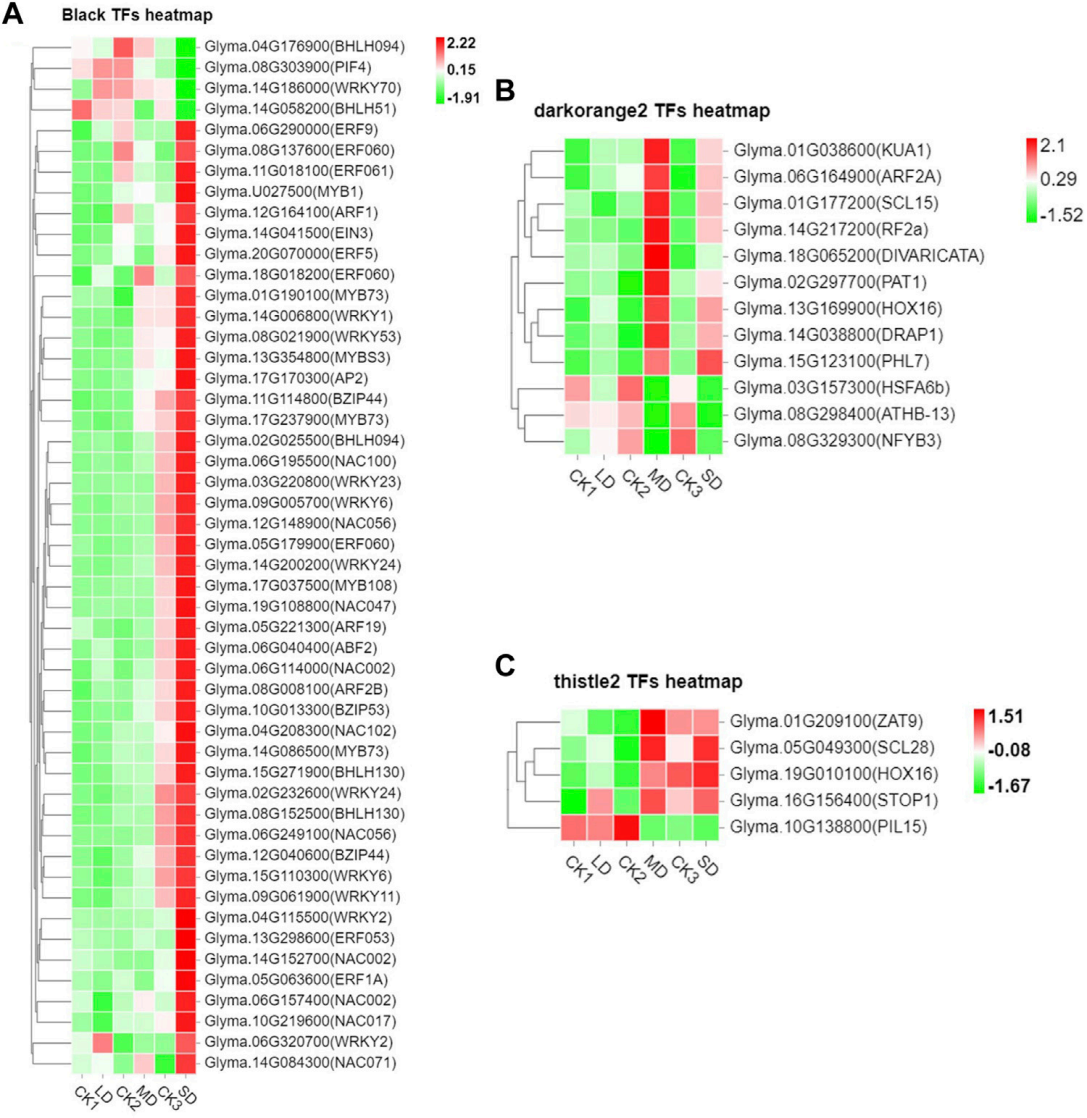
Heatmap of drought-induced TFs in three modules under drought stress. **(A)** Fifty drought-induced TFs in Black module. **(B)** Twelve drought-induced TFs in the Darkorange2 module. **(C)** Five drought-induced TFs in the Thistle2 module. The colors of the heatmap vary from green to red by normalizing the log_2_ (FPKM) of each gene.

We further performed weighted correlation network analysis based on antioxidant enzyme activity and osmotic regulation substance content to identify gene clusters or modules that are associated with the drought stress response in soybean leaves. All genes were clustered into 27 modules (Figure 3A), of which the black modules were significantly correlated (80%) with six physiological indicators (Figure 3B). The orange and tiles modules were also significantly correlated (Figure 3B). The gene expression trends for the three modules are shown in Figure 3C. These results indicate that the network of genes and these indicators is complex, and the functioning pathways are in these modules under drought stress. The KEGG enrichment analysis of three modules showed that the MAPK signaling pathway was enriched (black modules) for most drought-stressed samples, and ascorbate and aldarate metabolism and biosynthesis of secondary metabolites were also enriched (orange modules) (Supplementary Figure S6). In addition, the expression of 135 DEGs, 15 DEGs and 5 DEGs was associated with the TFs in the respective three modules (Supplementary Figure S7). Among them, the MYB (6), bHLH (5), bZIP (3), NAC (10), WRKY (11) and AP2/ERF (9) families of TFs were predominantly differentially expressed, and a greater number of TFs were significantly upregulated under MD and SD stress, such as *WRKY* (*Glyma.15G021900, Glyma.15G006800*), *MYB* (*Glyma.15G190100, Glyma.15G237900*), and *bZIP* (*Glyma.15G114800*) (Figure 4). Taken together, the drought response in soybean leaves was positively regulated by multiple metabolic pathways and a number of transcription factors.

### 3.5 DEGs involved in ABA biosynthesis and signaling pathways under drought stress

**FIGURE 5:**
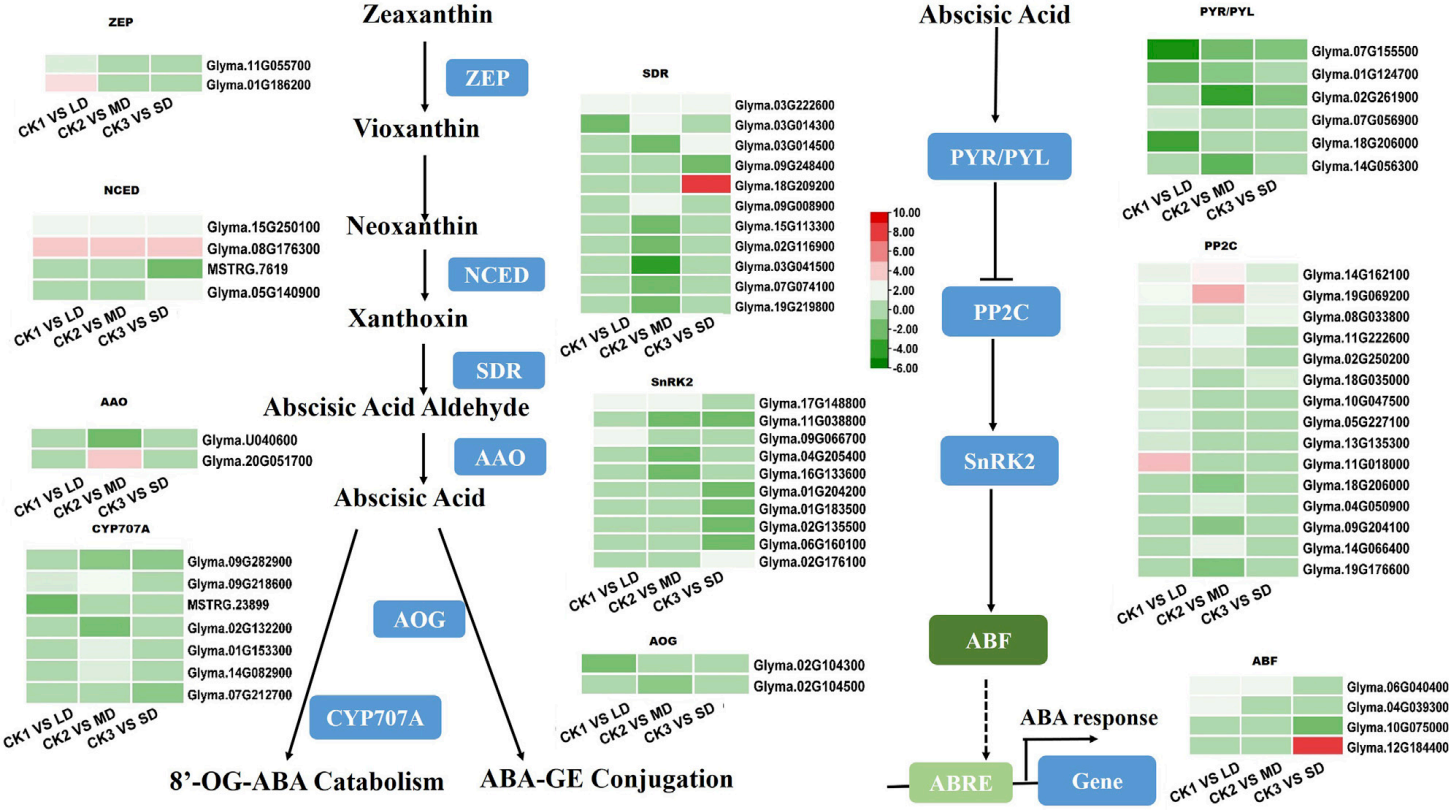
Heatmap of drought-responsive DEGs involved in ABA biosynthesis and signaling pathways under drought stress. Heatmap of drought-responsive DEGs involved in ABA biosynthesis and signaling pathways under drought stress. The colors of heatmap vary from green to red by normalizing the log_2_ (FPKM) of each gene.

The drought-responsive DEGs were significantly enriched in the plant hormone signal transduction pathways. As an important plant hormone, ABA plays an essential role in drought stress. Therefore, we monitored the expression patterns of ABA biosynthesis and its signaling related genes in the transcriptome data. Multiple genes that synthesize ABA were upregulated. Two ZEPs were upregulated only in LD treatment. At the same time, *NCEDs* (*Glyma.15G250100 and Glyma.08G176300*), the key genes in ABA synthesis, and *SDR* (*Glyma.03G222600*) were upregulated in all groups. *AAO* was only upregulated in the MD treatment. In addition, the metabolic genes AOG and *CYP707A* (*Glyma.09G282900*) were downregulated (Figure 5). Five ABA-induced PP2Cs were significantly upregulated by drought stress in the soybean, of which *Glyma.14G162100, Glyma.19G069200,* and *Glyma.08G33800* were upregulated under LD, MD and SD stress. In addition, three *SnRK2* genes (*Glyma.17G148800, Glyma.09G066700, Glyma.02G176100*) were upregulated, and six such genes were downregulated under MD or SD stress. Finally, three ABF TFs (*Glyma.06G040400* in LD and MD, *Glyma.04G039300* in LD, *Glyma.12G184400* in SD) were upregulated under three drought stress levels and in turn activated ABA-responsive response genes (Figure 5). This finding suggested that ABA-related genes are induced by varying degrees of drought stress and thus play important roles in the soybean response.

### 3.6 DEGs involved in AsA and GSH biosynthesis pathways under drought stress

**FIGURE 6:**
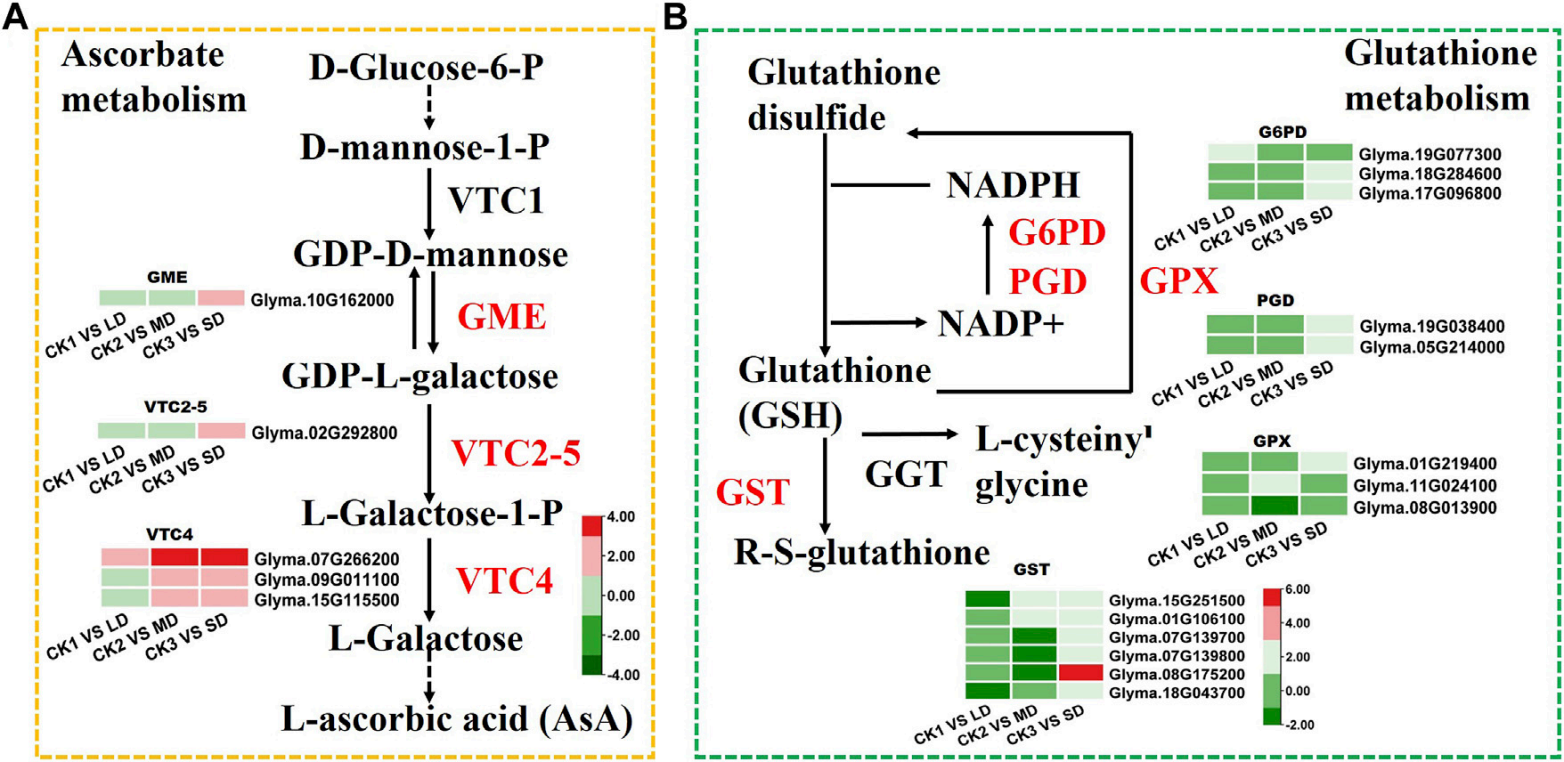
Heatmap of the DEGs involved in ascorbate and glutathione metabolism in response to drought stress. **(A)** The DEGs involved in ascorbate metabolism. **(B)** The DEGs involved in glutathione metabolism. The colors of heatmap vary from green to red by normalizing the log_2_ (FPKM) of each gene.

To identify the genes related to ascorbate biosynthesis and glutathione metabolism in soybean under drought stress, the DEGs related to ascorbate metabolism were analyzed (Figure 6). The six DEGs were found to be linked to ascorbate metabolism at all three drought stress levels. Some of these, DEGs, such as *GME* (*Glyma.10G162000*) and *VTC2-5* (*Glyma.02G292800*) were induced by SD stress and three *VTC4* genes (*Glyma.07G266200, Glyma.09G011100, Glyma.15G115500*) were upregulated under MD and SD stress (Figure 6A). Meanwhile, the DEGs were found to be related to glutathione metabolism under three levels of drought stress. Of these, six GST genes (glutathione S-transferase) and two *G6PDH* genes (*Glyma.18G284600, Glyma.17G096800*) were upregulated under SD stress, and some *GST* genes were downregulated under LD or MD stress. Furthermore, two *PGD* genes (*Glyma.19G038400, Glyma.05G214000*) and one *GPX* gene (*Glyma.01G219400*) were upregulated in the SD treatment (Figure 6B). These results showed that ascorbate and glutathione metabolism genes were activated by drought stress in soybean, especially by serious drought stress.

### 3.7 DEGs involved in flavonoid biosynthesis pathways under drought stress

**FIGURE 7:**
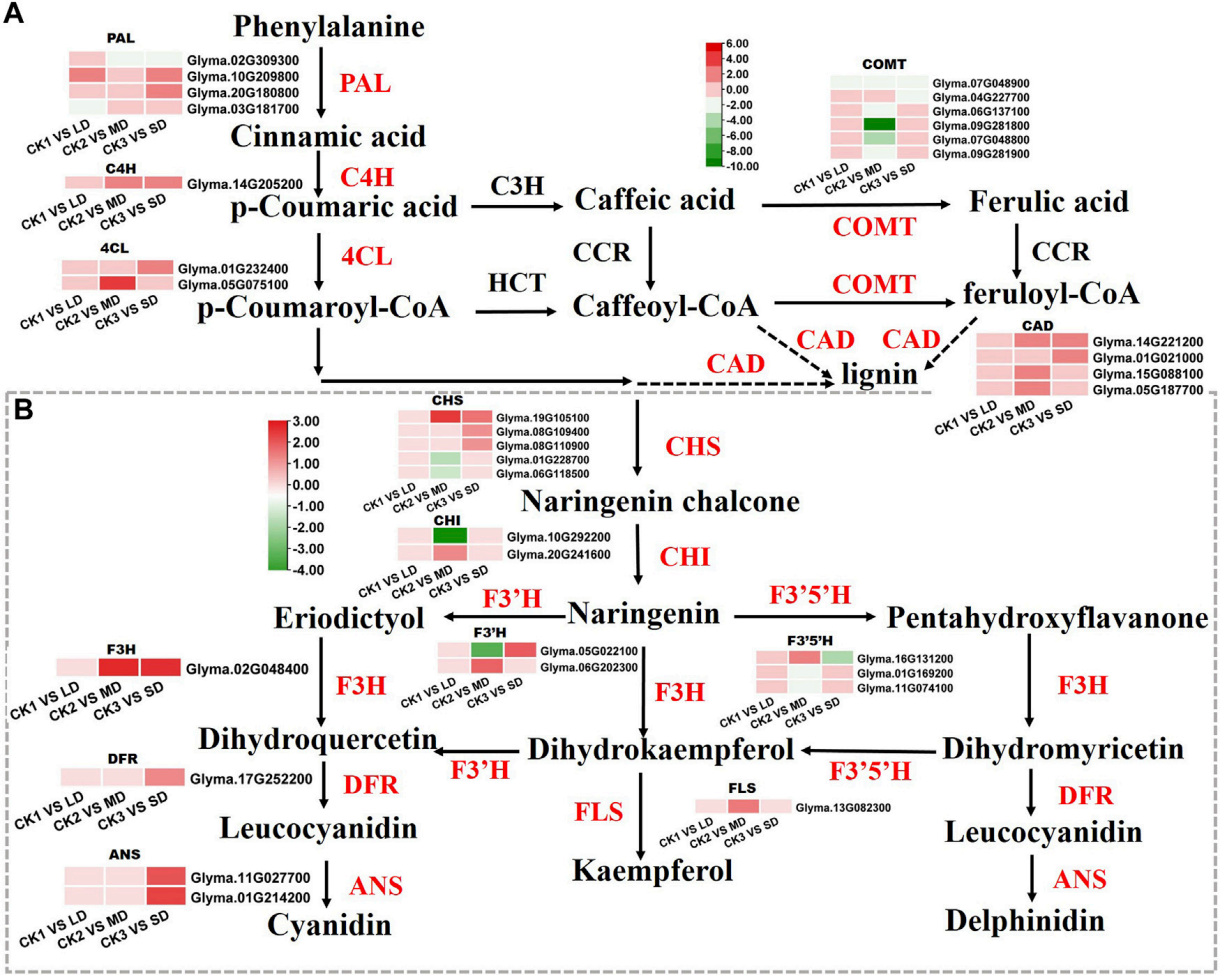
Heatmap of the DEGs involved in flavonoid biosynthesis in response to drought stress. **(A)** The DEGs involved in phenylalanine metabolism. **(B)** The DEGs involved in flavonoid biosynthesis. The colors of the heatmap vary from green to red by normalizing the log_2_ (FPKM) of each gene.

Based on the KEGG and WGCNA analyses, we further identified the DEGs involved in phenylalanine and flavonoid biosynthesis pathways, that were significantly related to drought responses in soybean leaves. We found that the DEGs enriched in phenylalanine and flavonoid biosynthesis pathways were unique under MD and SD treatments. Mild stress had no effects on phenylalanine and flavonoid biosynthesis pathways and was not linked to differentially expressed genes (Figure 7). Two genes (*Glyma.10G209800, Glyma.20G180800*) encoding PAL proteins involved in the phenylalanine pathway showed markedly increased expression in soybean leaves under LD or SD conditions relative to control plants. It is worth noting that *C4H* genes (*Glyma.14G205200*) and two *4CL* genes (*Glyma.01G232400, Glyma.05G075100*) were upregulated by MD and SD treatments (Figure 7A). Next, we further analyzed the genes of the flavonoid pathway under drought stress. The genes *CHS* (*Glyma.19G105100*) and *F3H* (*Glyma.02G048400*) were significantly upregulated under MD and SD stress. Meanwhile, *DFR* (*Glyma.17G252200*) and two *ANS* genes (*Glyma.11G027700, Glyma.01G214200*) were significantly upregulated under SD stress (up to 3-fold) (Figure 7B). These results suggest that the DEGs involved in the phenylalanine and flavonoid pathways are perhaps activated mainly by severe drought stress rather than mild drought stress.

### 3.8 The qRT-PCR analysis to validate RNA-seq data

**FIGURE 8:**
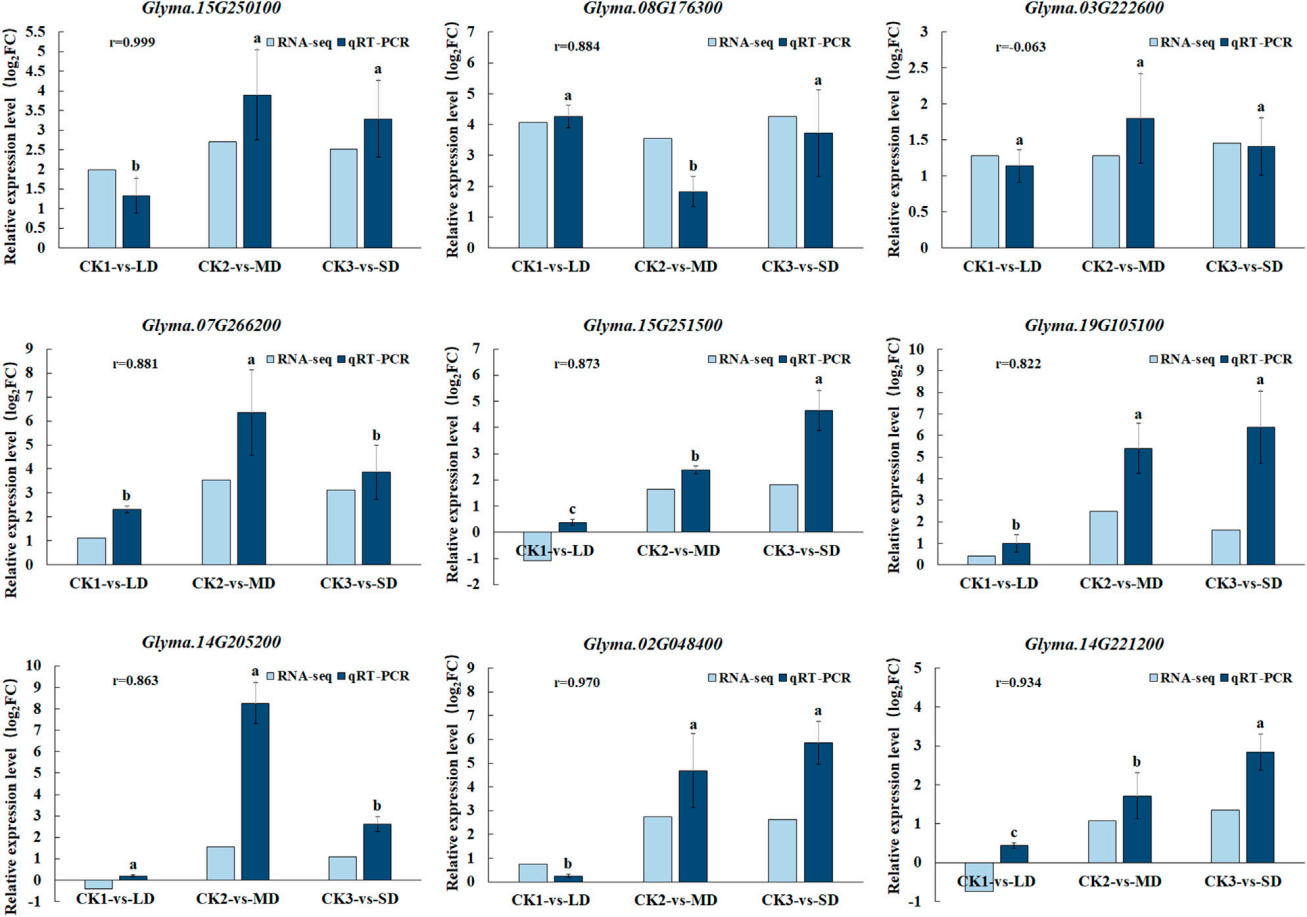
Comparison of gene expression patterns between RNA-seq and qRT-PCR under drought treatments. Data represent the means ± SD of three biological replicates, and each biological replicate contained three technical replicates (*p* < 0.05).

To validate the gene expression data obtained from RNA-seq, we selected nine genes participated in ABA, AsA, GSH and flavonoid biosynthesis pathways under drought stress for qRT-PCR analysis. We found good agreement (r = 0.99–0.8) in with relative gene expression between RNA-Seq and qRT-PCR for all candidate genes except *Glyma.03G222600* under drought stress, which confirmed the reliability and accuracy of RNA-seq analyses in this study (Figure 8). When the soil moisture content was 20.3% (water withheld for 17 days), the upregulated genes (*Glyma.15G250100, Glyma.07G266200* and *Glyma.14G205200*) in the MD treatment increased by the largest proportion, to 3.7-, 6.5- and 7.6 fold, respectively. In turn, the up-regulated genes (*Glyma.15G251500, Glyma.02G048400, Glyma.19G105100 and Glyma.14G221200*) exhibited the largest increase (to 3.7-,6.5- and 7.6- fold) at SD treatment after 27 days without water, at which time the soil moisture content was 16.9%. Overall, when soybean exposed to MD and SD treatments, some genes involved in ABA, AsA, GSH and flavonoid biosynthesis pathways in soybean leaves were highly expressed, and as the water deficit treatment was extended, the degree of gene expression increased correspondingly.

### 3.9 Soybean yield after rehydration

**FIGURE 9:**
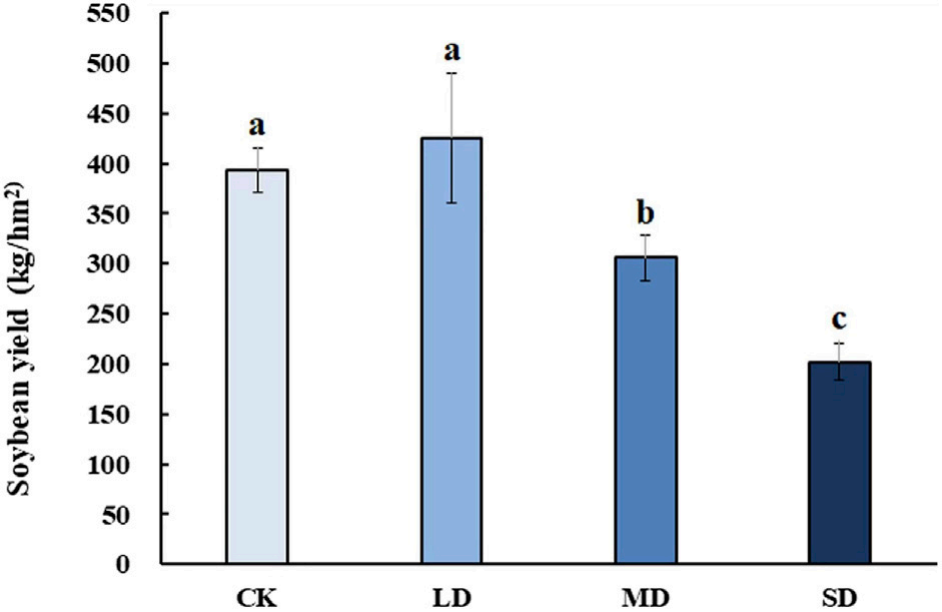
The effect of varying degrees of drought stress on soybean yield after rehydration. Three biological replicates were performed and the data represent the means ± SDs. Different letters represent statistically significant differences (*p* < 0.05).

To reveal the effect of varying degrees of drought stress on the yield of soybean after rehydration, soybean was harvested at the mature stage for analysis**.** Soybean yield significantly decreased with drought stress at different levels, whereas mild drought stress had no significant effect on soybean yield after rehydration in the harvested period (*p* < 0.05) (Figure 9). Moreover, soybean yield under MD and SD stress was approximately 0.75-and 0.5- fold lower than that of the controls, respectively (*p* < 0.05).

## 4 Discussion

As a symptom of a severe global climate disaster, drought leads to a decline in crop production and exacerbates direct economic losses each year (Molnár et al., 2021). In soybean, which is a global economic oilseed crop, drought stress is a major factor that can reduce yields by more than 40% (Specht et al., 1999). However, molecular insights into the drought resistance of soybean have been limited.

### 4.1 Physiological performance of soybean leaves subjected to different levels of drought stress

Plants have been shown to display a variety of changes to reduce drought damage through at stress response mechanisms in terms of physiology and biochemistry (Harauma et al., 2007; Song et al., 2009; Elansary and Salem, 2015; Martignago et al., 2020). In this study, mild drought stress had little effect on the growth of soybean, however, the leaves wilted, curled, and dehydrated dramatically under moderate and severe stress levels. The phenotype of plant leaves directly indicates the degree of drought stress (Li et al., 2020). When plants encounter drought stress, excess reactive oxygen species (ROS) will accumulate, and these will be scavenged by activating the antioxidant system to reduce oxidative damage, including POD, SOD and CAT activities (Miller et al., 2010). If the antioxidant system cannot remove excess reactive oxygen species in time, they will cause damage to membranes and protein function, thereby harming plant tissues and accelerating plant senescence (Mittler et al., 2004; Torres and Dangl., 2005). One of the associated damages to plants is the production of MDA and membrane lipid peroxidation (Gill and Tuteja, 2010). Our findings suggested that MDA content was increased, which was caused by ROS accumulation under drought stress (Figure 1B). The activities of SOD and CAT were successfully measured, and they were found to be upregulated to remove excess ROS and relieve membrane damage to maintain normal plants growth (Figure 1B) (Baldoni et al., 2016). Plants also adopt osmotic regulation strategies to confer drought tolerance and further reduce the damage caused by drought stress (Lotfi et al., 2010). Notably, soluble proteins, soluble sugars and proline increased approximately 2-fold under SD stress, which contributed to reducing the osmotic potential and membrane protection, as well as maintaining water uptake in plants in response to drought stress (Ashraf and Foolad, 2007; Seki et al., 2007)**.**

### 4.2 The roles of ABA biosynthesis and signaling and TFs in drought stress responses in soybean

ABA plays a pivotal role in drought stress tolerance that results in closing the stomata as well as regulating the contents of proteins, soluble sugars and amino acids (Finkelstein, 2013; Maruyama et al., 2014; Gonz´alez-Villagra et al., 2019; Mathan et al., 2021). Using GO and KEGG enrichment analyses, 28 DEGs and 36 DEGs related to ABA biosynthesis and signaling pathways in soybean under different drought treatments were obtained (Figure 5). We found that the highest expression of the NCED gene occurred in soybean under three drought stress levels (Figure 5A), which demonstrated that the plant’s ‘commitment’ increased ABA content (Qin and Zeevaart., 2002; Martínez-Andújar et al., 2011). Interestingly, it seems that the expression of *AOG* and *CYP707A* genes was significantly lower than that of the control after MD and SD treatments (Figure 5A). This suggested that the ABA response to drought stress occurs through increased biosynthesis and catabolism (Ren et al., 2006). This phenomenon was also reflected at the transcription levels of ABA signaling pathways under drought stress. Drought treatments upregulated genes participating in the ABA signaling pathways, especially three *PP2C* genes (Figure 5B). Notably, we observed that the expression of three *ABF* genes in soybean treated with drought stress was higher than that in the controls. These results are compatible with the critical signaling role of ABA in plant defense.

Transcription regulation is a typical mechanism for plants encountering drought. Based on WGCNA, *MYB, WRKY, NAC*, and *bHLH* were upregulated under MD and SD stress (Figure 3). They function as activators or repressors to regulate target genes and then form a transcriptional regulatory network involved in abiotic stress response and tolerance. In addition, some plant hormone response factors were also enriched in the three modules, such as *ARF*, *EIN3* and *BES1*, which indicated that multiple hormones co-regulate plant responses to drought stress.

### 4.3 AsA and GSH metabolism involved in drought stress responses in soybean

When the plant is subjected to drought stress, the excess ROS will be scavenged by the antioxidant system, which involves the SOD, POD and CAT enzymes, as well as the nonenzymatic constituents **(**AsA, GSH, and flavonoids) that maintain the steady state of cell membranes (You et al., 2019). KEGG analysis detected the enrichment of AsA and GSH metabolism genes involved in the drought response (Figures 2C–F; Figure 6). Vitamin C, also known as l-ascorbic acid (AsA), protects cells from oxidative stress by maintaining the ROS balance (Gallie, 2013; Akram et al., 2017). The Smirnoff-Wheeler pathway (*VTC1, GME, VTC2, VTC4* and *VTC5*) was identified to participate in vitamin C biosynthesis (Wheeler et al., 1998; Conklin et al., 1999, 2000, 2006; Dowdle et al., 2007). The relevant pathway genes of AsA metabolism were all upregulated in both the MD and SD treatments, especially the VTC4 genes under SD stress. A recent study indicated that the molecular mechanism underlying AsA biosynthesis and ABA signaling pathways participates in plant drought tolerance (Zhang et al., 2020). The application of AsA can be used against drought stress in various plant species, such as wheat (Malik and Ashraf., 2012; Hussein et al., 2014), and maize (Darvishan et al., 2013). In short, these results provide evidence that AsA biosynthesis was significantly induced by drought stress in soybean, especially by severe drought stress.

An additional mechanism by which plants increase drought resistance is the accumulation of glutathione, a major antioxidant that conjugates with electrophilic compounds and facilitates peroxide reduction (Alscher, 1989; Anderson and Davis, 2004). Specifically, this involves upregulating G6PDH and GST enzymes involved in scavenging ROS and reducing secondary noxious products under drought stress (Wagner et al., 2002; Anderson and Davis, 2004; Landi et al., 2016). In the present study, two *G6PDH* and six *GST* genes were upregulated by approximately 2-fold under SD treatment, indicating that soybean can activate the *GSH* metabolism pathway to scavenge ROS under SD stress. Previous studies also reported that increasing *G6PDH* expression in the tomato and the overexpression of *GST* genes in transgenic *Arabidopsis* can enhance drought tolerance (Liu et al., 2013; Xu et al., 2015).

### 4.4 Flavonoid biosynthesis involved in drought stress responses in the soybean

Flavonoids are widely distributed in plants and exhibit antioxidant activities, which could increase the ROS scavenging ability to protect normal plant growth from drought stress (Falcone Ferreyra et al., 2012; Nakabayashi et al., 2014). Flavonoids are synthesized by the phenylalanine pathway in plants (Dixon et al., 2002). The C4H pathway is involved in the drought defense of cucumber (Bellés et al., 2008). In our study, severe drought stress significantly upregulated the transcript abundance of *C4H* genes and activated the transcription of two *4CL* genes (Figure 7A). We further detected that the transcription of four CAD genes (*Glyma.14G221200, Glyma.01G021000, Glyma.15G088100, Glyma.05G187700*) was markedly increased, which may be involved in lignin synthesis to confer drought tolerance to soybean (Xu et al., 2020).

Earlier research analyzed the key genes related to flavonoid biosynthesis by transcript profiling under drought stress (Kang et al., 2011). Similarly, the DEGs of flavonoid biosynthesis pathway were activated by drought stress in *Populus euphratica* (Jiao et al., 2021). The KEGG analysis in our study established that the DEGs were enriched in flavonoid metabolism biosynthesis only under MD and SD stress (Figure 2). The key genes *CHS, F3H, DFR* and *ANS* showed prominent increases under SD stress, which may have elevated the contents of flavonoids and mobilized them in response to drought resistance (Figure 7). These results indicate that drought stress regulated the synthesis of flavonoids, which played a key role in enhancing drought tolerance in soybean.

### 4.5 A proposed model and the effect of drought stress on soybean yield

Plant growth compensation by rehydration is one of the strategies against short-term drought to alleviate its effect on plant yield (Acevedo et al., 1971), which was also performed in this study. Short periods of drought had no effect on the soybean yield, but prolonged periods of drought reduced the yields by 50%.

**FIGURE 10:**
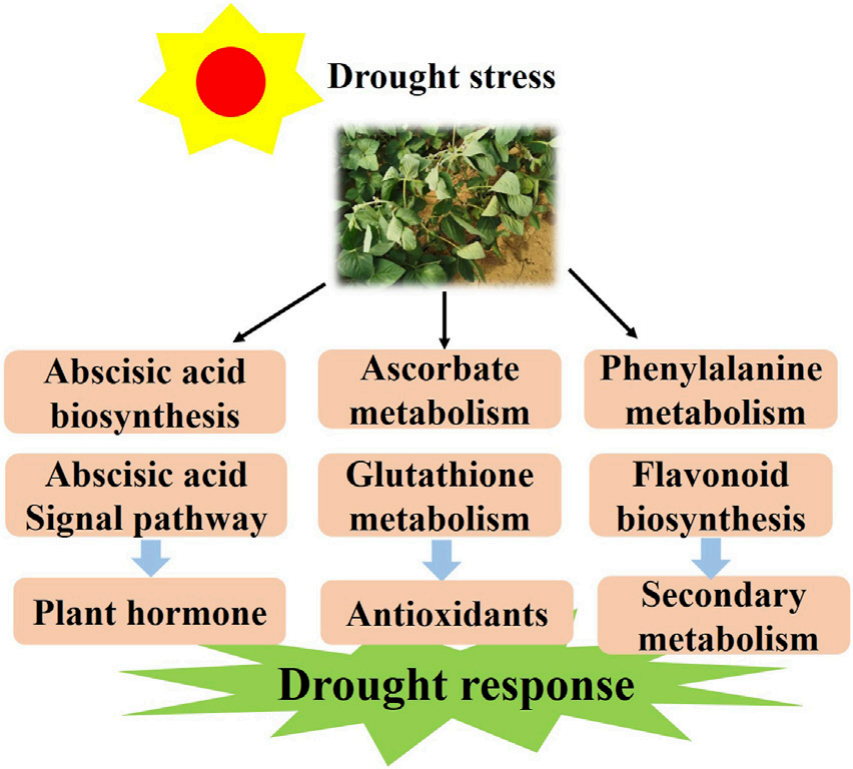
The model for the regulation of the drought response in soybean responds to drought conditions by regulating the plant hormone, antioxidant and flavonoid pathways.

It is known that half of the drought response genes are activated by ABA (Seki et al., 2002). However, the molecular mechanism induced by drought stress in soybean remains elusive and needs to be further studied. Herein, we discuss the probable molecular mechanism of soybean response to drought stress. According to the results, we propose a model for the soybean response to varying levels of drought stress. First, drought stress activates the ABA biosynthesis and signaling and drought-related TFs in plants. Meanwhile, it promotes AsA and GSH metabolism by regulating the expression of the *VTC, GME* and *G6PDH* genes. With longer time exposure to drought stress, flavonoids may be a positive regulator and function to mediate drought resistance (Figure 10). The above results not only deepen the insights into the drought response in soybean but also provide a theoretical basis for the genetic improvement and water-efficient irrigation of this crop.

## 5 Conclusion

In this study, the response mechanism of soybean exposed to three levels of drought stress at the flowering stage was investigated. Physiological, transcriptomic and WGCNA analyses were carried out and the TFs and potential pathways of genes in soybean under drought stress were identified. The results suggested that the soybean plant is able to activate the genes of antioxidants, secondary metabolism and hormone signaling pathway, *Glyma.08G176300(NCED1), Glyma.03G222600(SDR), Glyma.02G048400(F3H), Glyma.14G221200(CAD), Glyma.14G205200(C4H) and Glyma.19G105100 (CHS), Glyma.07G266200 (VTC5-2) and Glyma.15G251500 (GST)*, response to drought stress by promoting MDA accumulation and the activities of SOD and CAT to partly cope with drought stress. Furthermore, the soybean yield after rehydration in the harvesting period was reduced by 50% under severe drought stress. Finally, we further deepen the understanding of molecular mechanism of soybean in response to drought stress, which provides a theoretical basis for the molecular breeding of drought resistance.

## Data Availability

The data presented in the study are deposited in the NCBI SRA repository, accession number PRJNA852689.
