# Supplementary figures and images for: Transcriptome analysis reveals key drought-stress-responsive genes in soybean
Source: Front Genet. 2022 Nov 28;13:1060529. doi: 10.3389/fgene.2022.1060529 (PMC9742610; doi:10.3389/fgene.2022.1060529)

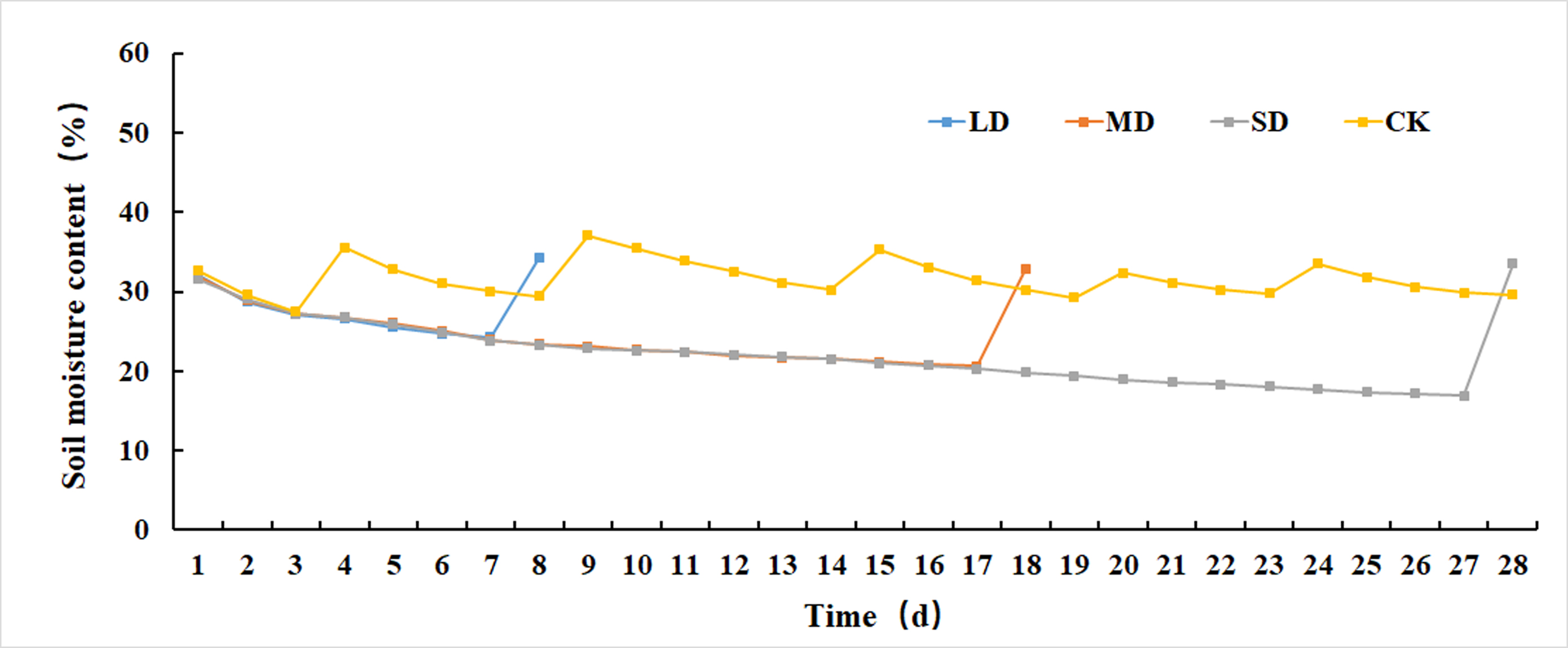


**Fig. S1.**


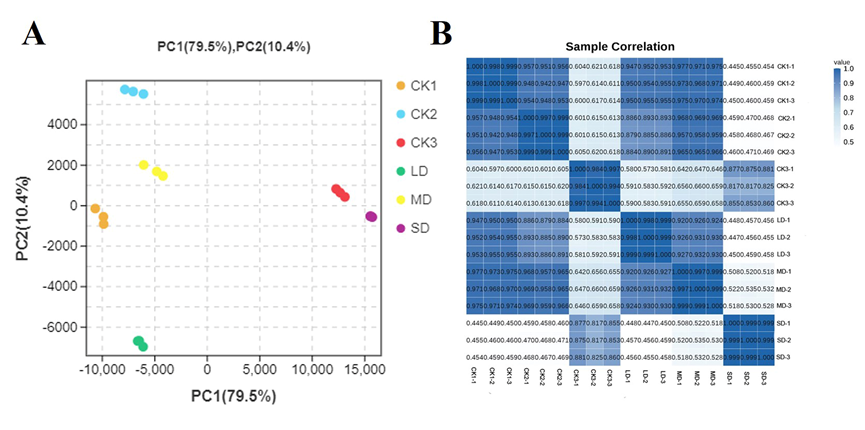


**Fig. S2.**

**
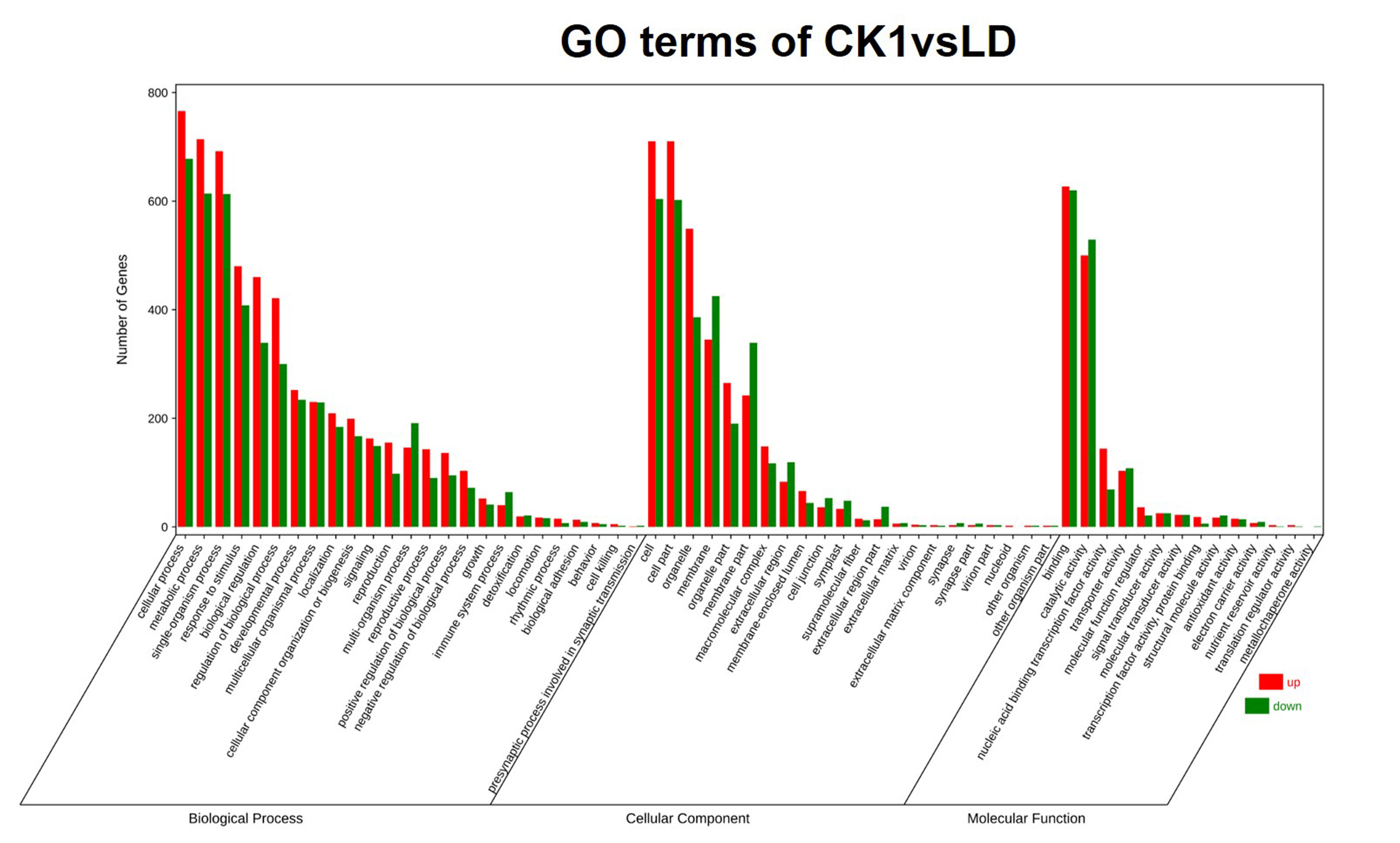
**

**Fig. S3.**

**
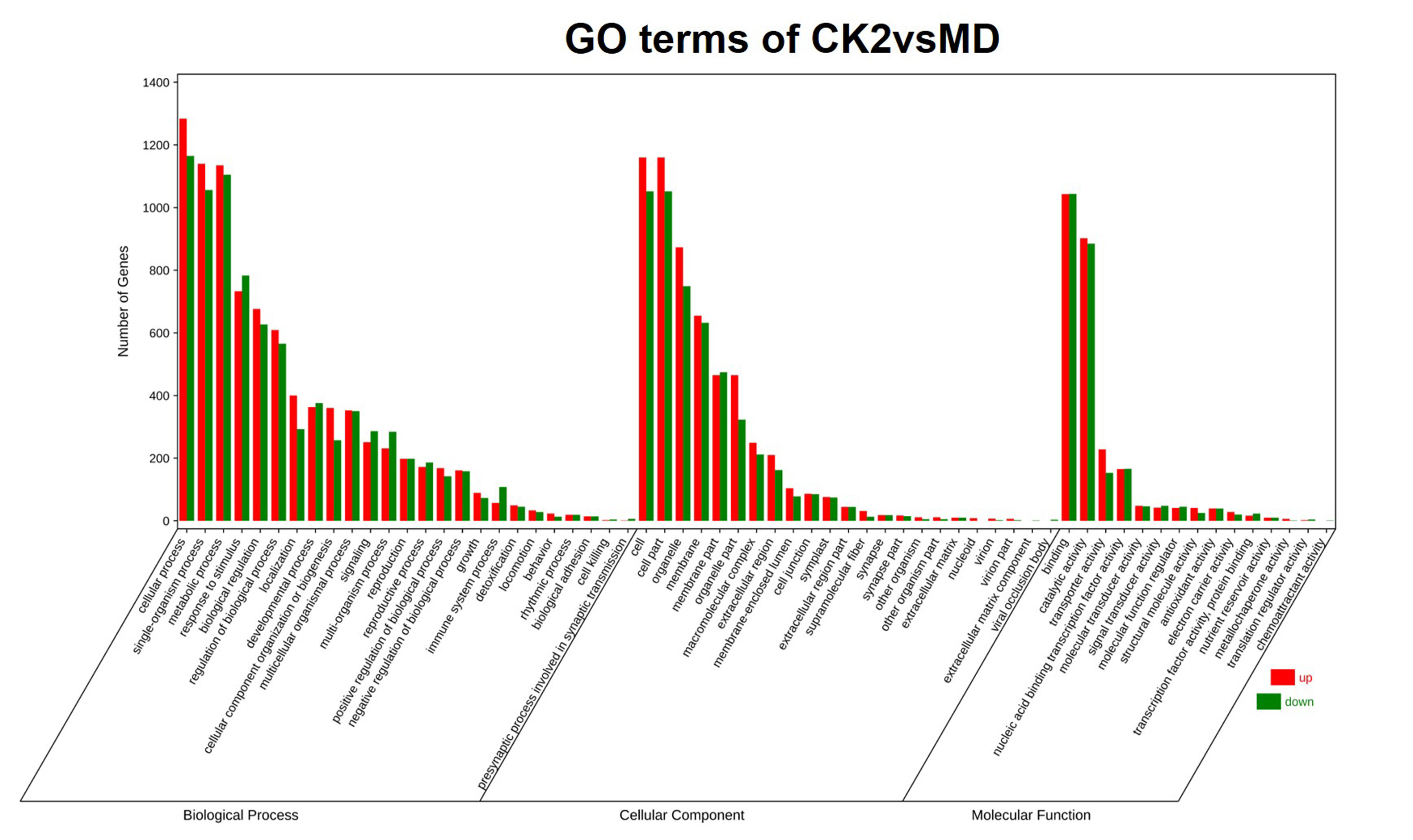
**

**Fig. S4.**


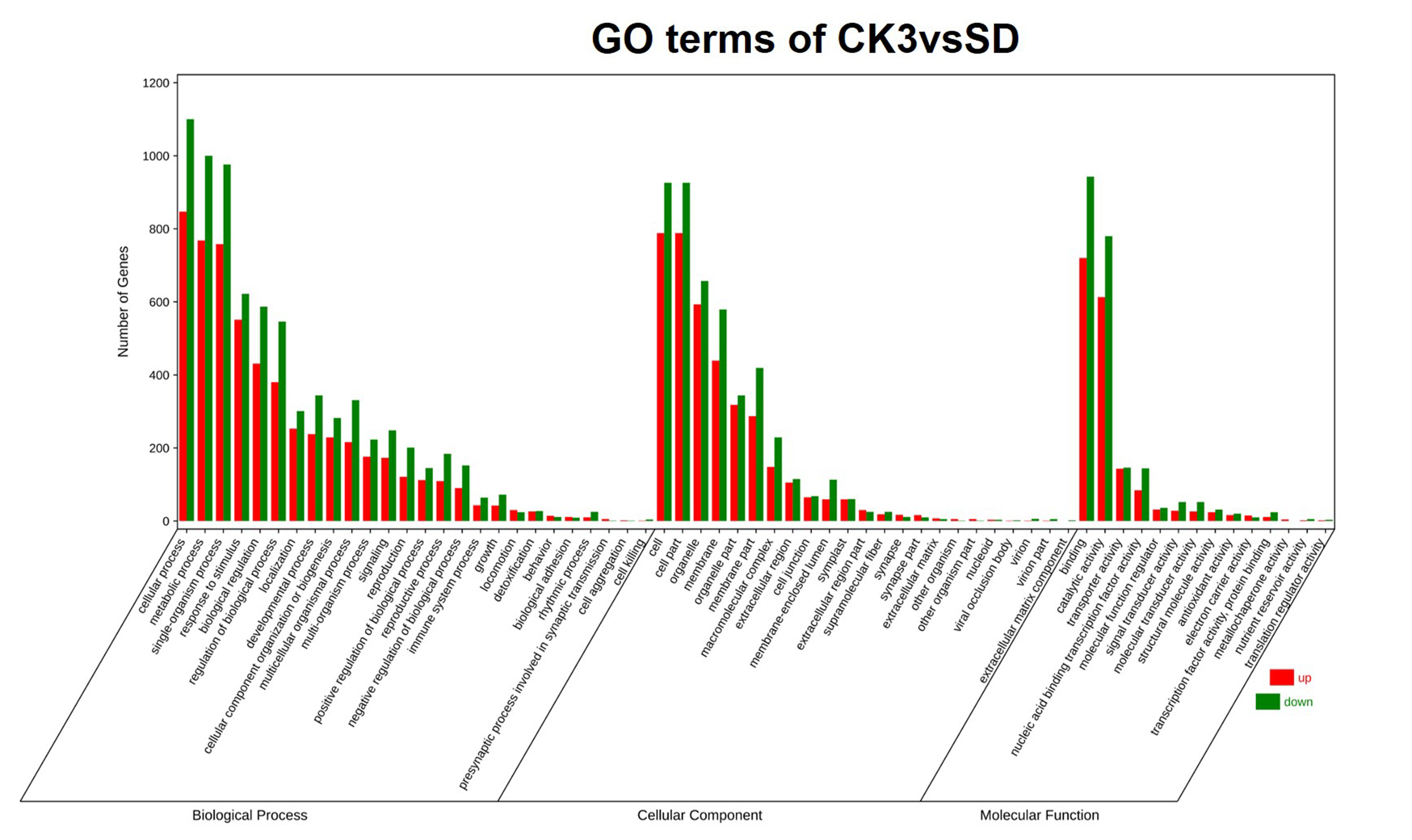


**Fig. S5.**


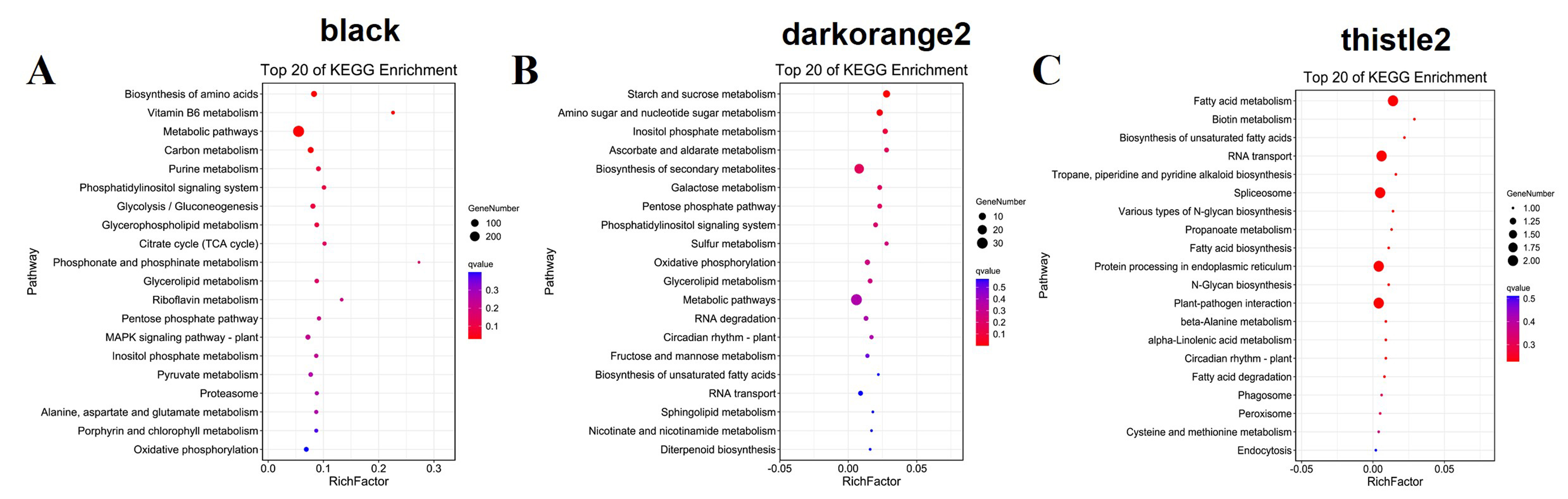


**Fig. S6.**


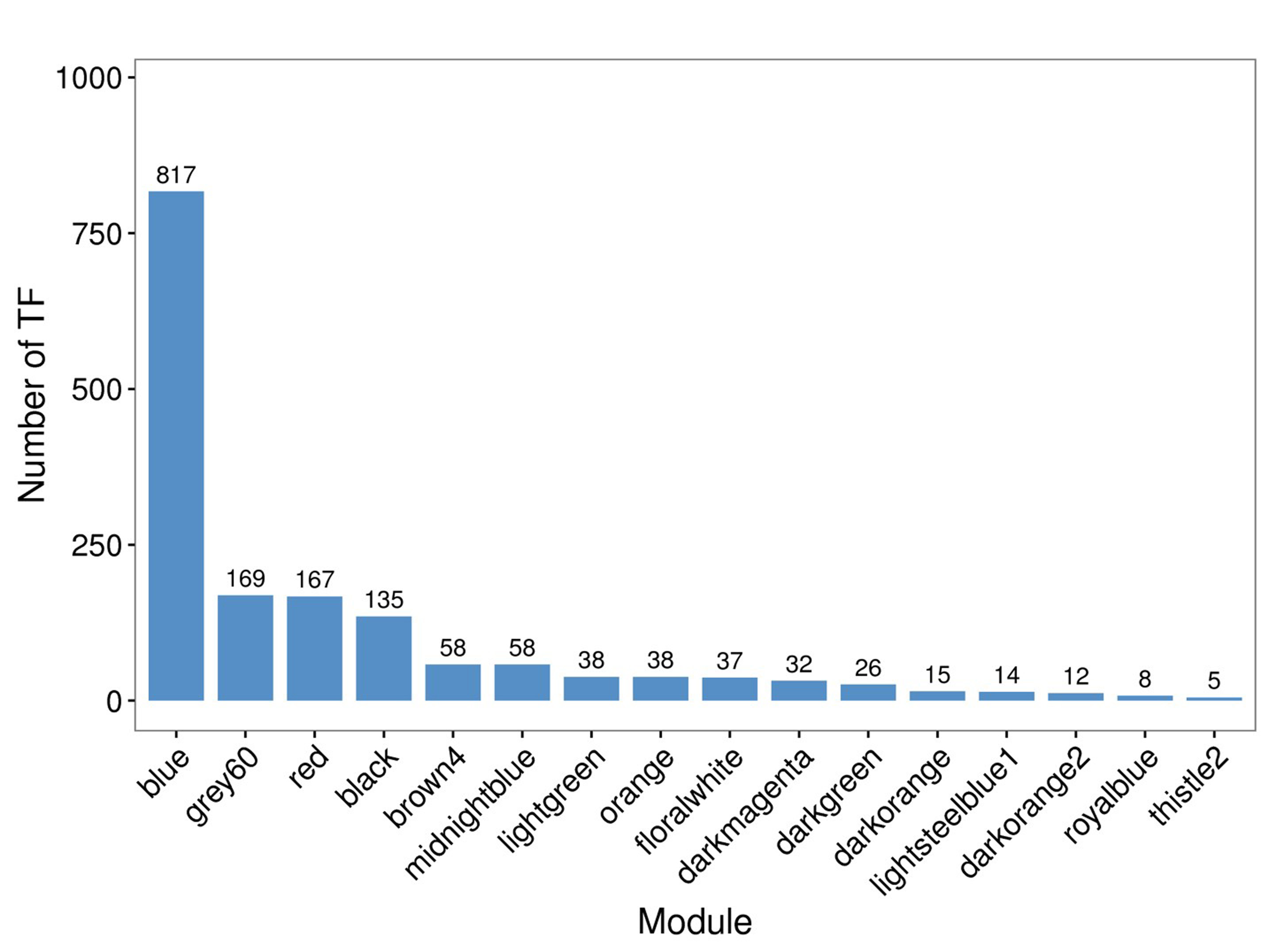


**Fig. S7**

Supplement: Supplementary file 1 [file DataSheet1.docx]
